# The visually estimated blood volume in scaled canisters based on a simulation study

**DOI:** 10.1186/s12871-021-01265-1

**Published:** 2021-02-16

**Authors:** Lara Gerdessen, Vanessa Neef, Florian J. Raimann, Kai Zacharowski, Florian Piekarski

**Affiliations:** Department of Anaesthesiology, Intensive Care Medicine and Pain Therapy, University Hospital Frankfurt, Goethe University, Frankfurt, Theodor-Stern-Kai 7, 60590 Frankfurt am Main, Germany

**Keywords:** Blood loss estimation, Visual estimation, Transfusion, Patient blood management

## Abstract

**Background:**

The most common technique used worldwide to quantify blood loss during an operation is the visual assessment by the attending intervention team. In every operating room you will find scaled suction canisters that collect fluids from the surgical field. This scaling is commonly used by clinicians for visual assessment of intraoperative blood loss. While many studies have been conducted to quantify and improve the inaccuracy of the visual estimation method, research has focused on the estimation of blood volume in surgical drapes. The question whether and how scaling of canisters correlates with actual blood loss and how accurately clinicians estimate blood loss in scaled canisters has not been the focus of research to date.

**Methods:**

A simulation study with four “bleeding” scenarios was conducted using expired whole blood donations. After diluting the blood donations with full electrolyte solution, the sample blood loss volume (SBL) was transferred into suction canisters. The study participants then had to estimate the blood loss in all four scenarios. The difference to the reference blood loss (RBL) per scenario was analyzed.

**Results:**

Fifty-three anesthetists participated in the study. The median estimated blood loss was 500 ml (IQR 300/1150) compared to the RBL median of 281.5 ml (IQR 210.0/1022.0). Overestimations up to 1233 ml were detected. Underestimations were also observed in the range of 138 ml. The visual estimate for canisters correlated moderately with RBL (Spearman’s rho: 0.818; *p* < 0.001). Results from univariate nonparametric confirmation statistics regarding visual estimation of canisters show that the deviation of the visual estimate of blood loss is significant (z = − 10.95, *p* < 0.001, *n* = 220). Participants’ experience level had no significant influence on VEBL (*p* = 0.402).

**Conclusion:**

The discrepancies between the visual estimate of canisters and the actual blood loss are enormous despite the given scales. Therefore, we do not recommend estimating the blood loss visually in scaled suction canisters. Colorimetric blood loss estimation could be a more accurate option.

## Introduction

The quantification of blood loss is essential for intraoperative management and plays a key role in transfusion decision making [1]. The visual assessment by intervention team is the most common technique used worldwide to quantify blood loss during an operation. This does not only include the estimation of blood volume in surgical drapes and suction canisters, but also the recording of external blood loss. However, it is known that this method is associated with systematic errors (under- or overestimation of blood loss) depending on the person making the estimation [2, 3]. In every operating room you will find scaled suction canisters that collect fluids from the surgical field. This scaling is regularly used by clinicians for visual assessment of blood loss. While many studies have been conducted to quantify and improve the inaccuracy of the visual estimation method, research has focused on the estimation of blood volume in surgical drapes [4–6]. The question whether and how scaling of canisters correlates with actual blood loss and how accurately clinicians estimate blood loss in scaled canisters has not been the focus of research to date. This simulation study at a German university hospital examines the accuracy with which anesthetists estimate blood loss in scaled canisters. The aim of the present study was to evaluate the difference between the reference blood volume and the visually estimated quantity in canisters.

## Material and methods

This study was approved by the Ethics Committee (IRB) at the University Hospital Frankfurt, Goethe University (Ref: 163/19) and conducted in accordance with the Helsinki Declaration. Participation was voluntary and each participant gave their written consent.

The purpose of this study was to assess the deviation from the reference volume in the estimation of visual blood loss in scaled canisters by anesthetists.

### Structure of the simulation

For the simulation, four scenarios were set up. Each scenario consisted of one scaled canister placed on a blanket on the floor or Table. A wall separated the scenarios from each other. The participants were anesthetists with various levels of experience. Per scenario all participants had 90 s to record the visually estimated (V-EBL) blood loss per canister in milliliter and document their estimation in a case report form (CRF). Each scenario was assessed individually by each participant. All scenarios were presented simultaneously to the participants in the form of a parcourse. After 90 s, participants were prompted by a signal tone to switch to the next scenario.

The participants were requested to specify the estimated volume per canister as if the situation were real. To avoid manipulating the participants’ responses, no additional case information were provided. The trial was performed under bright, operating room-like lighting conditions (median 882 lx). The lighting conditions were measured with a luxmeter (TFA Dostmann LM37 luxmeter, TFA Dostmann GmbH & Co. KG, Wertheim-Reicholzheim, Germany).

**Fig. 1:**
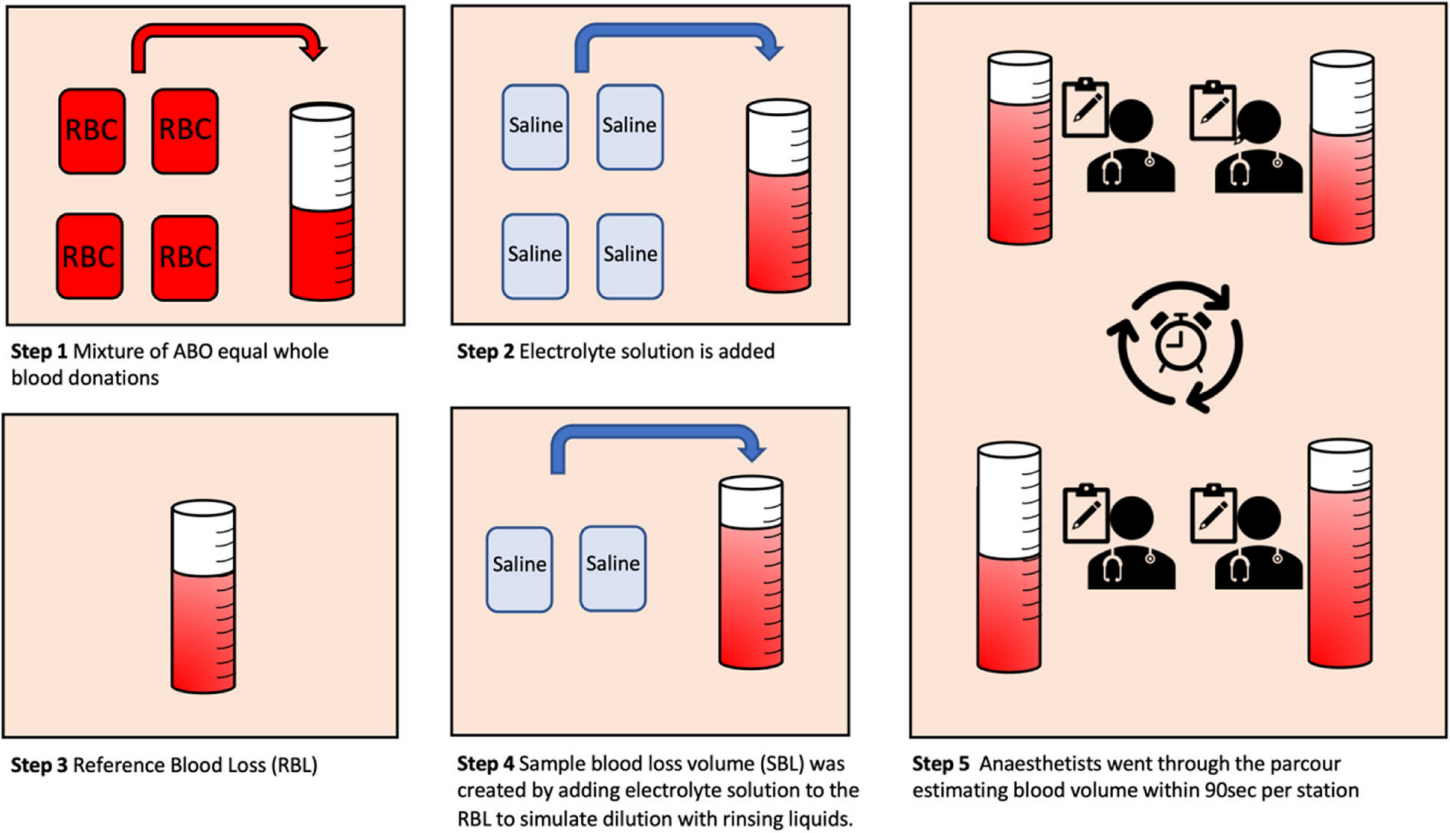
Illustration of the experimental setup and production of the reference blood loss (RBL)

For the experimental setup, expired or unusable whole blood donations (provided by the German Red Cross, Institute for Transfusion Medicine, Goethe University Frankfurt, Germany) were diluted with whole electrolyte solutions (Sterofundin ISO, B. Braun, Melsungen, Germany) to generate predetermined volumes (188 ml–1267 ml) with defined hemoglobin (Hb) values (9.5–12.1 g/dl). The use of whole blood donations is a well-established method for the experimental determination of blood loss [2]. The prepared mixture was set as the reference blood loss (RBL). The Hb level was measured by blood gas analysis (Radiometer ABL800 Flex, Radiometer GmbH, Krefeld, Germany) after each step. In order to simulate typical dilution effects by irrigation, ascites or liquid therapy with crystalloids, fully electrolytic solution was added to the RBL. This mixture was defined as sample blood loss volume (SBL) with varying hemoglobin concentrations (4.9–6.2 g/dl) (Fig. 1). Table 1 shows the breakdown of each scenario by RBL, Hb and Hematocrit (Hct) levels, dilution, and total volume in the canister.

**Table 1:** Breakdown of the different scenarios

		Scenario 1	Scenario 2	Scenario 3	Scenario 4
Reference blood loss (RBL)	Volume (ml)	1267	287	188	276
	Hb (g/dl)	11.7	9.5	12.1	10.0
	Hct (%)	36.0	29.4	37.2	31.0
Electrolyte solution added to RBL	Volume (ml)	633	323	137	209
Sample blood loss (SBL)	Volume (ml)	1900	610	325	485
	Hb (g/dl)	6.4	6.2	6.0	4.9
	Hct (%)	20.0	19.6	19.0	15.4

The volumes, hemoglobin (Hb) and hematocrit (Hct) levels of sample preparation are shown. The reference blood loss (RBL) was diluted with electrolyte solution to simulate dilution with rinsing liquids. The resulting sample blood loss volume (SBL) was then filled into the canisters and estimated by the anesthetists

At the blood donation center, the blood donations were treated beforehand routinely with CPD stabilizer solution to prevent blood clotting. The CPD solution consists of citrate buffer, sodium dihydrogen phosphate, glucose and adenine. For this reason, we have not added an additional anticoagulant to the RBL or SBL.

Each canister was prepared with a predefined volume (325 ml–1900 ml) of SBL. A different SBL was used in each scenario. The scenarios were presented in a randomized order to the participants.

The following canisters were used: Serres, suction canister, 3000 ml with pre-gelled bag, folded (Serres Oy, Kauhajoki as. Finland). The influence of the gel on volume expansion was tested in advance and ruled out. To simulate a situation, close to real conditions, the canisters were filled under vacuum.

### Statistics

A priori analysis was conducted to calculate the sample size. With 90% power, a significance level of 0.05, and Cohen’s d of 0.5, a minimum of 44 participants was calculated.

Descriptive statistics were performed using IBM SPSS® Statistics (Version 26, IBM®, Armonk, New York, USA) and Microsoft® Office Excel (Mac Version 16.3, Microsoft Corporation, Redmond, Washington, USA).

Variables are expressed in mean (95% confidence interval, CI), median (25/75 IQR, interquartile range) or count (%, percentage) as appropriate. A concordance analysis was performed using the Bland-Altman framework for agreement between two measurements. Spearman’s rank correlation coefficient was calculated for comparison of V-EBL. Univariate nonparametric confirmation statistics with paired Wilcoxon test were performed. A *p* value of 0.05 or less was considered to be statistically significant.

## Results

**Fig. 2:**
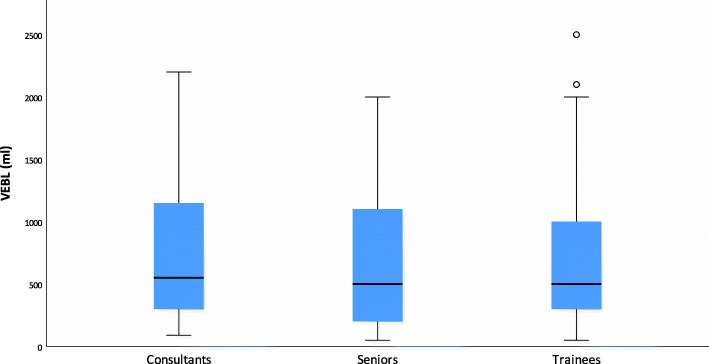
Boxplots for V-EBL for different experience levels Boxplots show estimated visual blood loss (V-EBL) for different experience levels

**Fig. 3:**
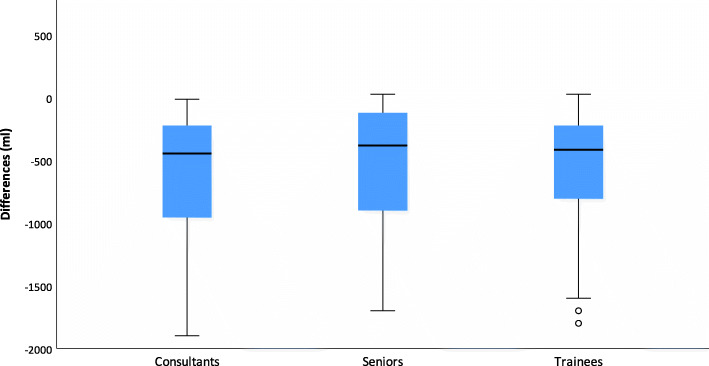
Boxplots for the difference for different experience levels Boxplots show differences between estimated visual blood loss (V-EBL) and reference blood loss (RBL) for different experience levels

Fifty-three anesthetists participated in this study. All 53 CRFs were completed and analyzed. The educational level of the participants was divided as follows: Anesthesia trainees (52%), specialists (25%) and senior physicians (23%). Three years was the average clinical work experience for the assistant physicians, seven years for the specialists and 15 years for the senior physicians. There was no significant effect of the participants’ level of experience regarding the VEBL (*p* = 0.402) and the difference from RBL (*p* = 0.364) (Figs. 2 and 3).

**Fig. 4:**
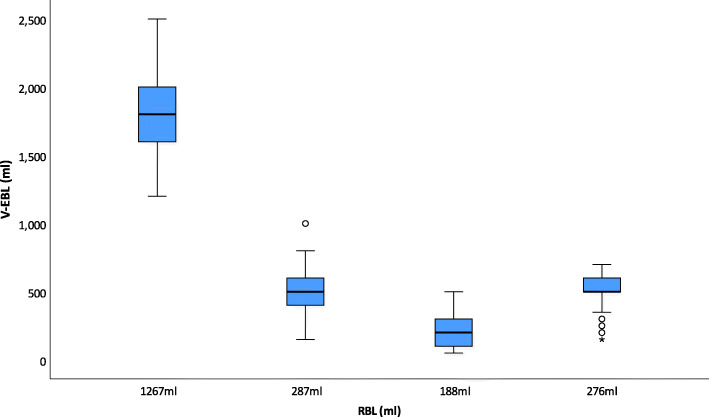
Boxplots for V-EBL in canisters Boxplots show estimated visual blood loss (V-EBL) and the reference blood loss (RBL) for canisters

The median estimated blood loss was 500 ml (IQR 300/1150) compared to the RBL median of 281.5 ml (IQR 210.0/1022.0). Figure 4 shows the V-EBL in canisters and the deviation from the RBL per scenario. Overestimations up to 1233 ml as well as underestimations with a range of 138 ml were observed.

**Fig. 5:**
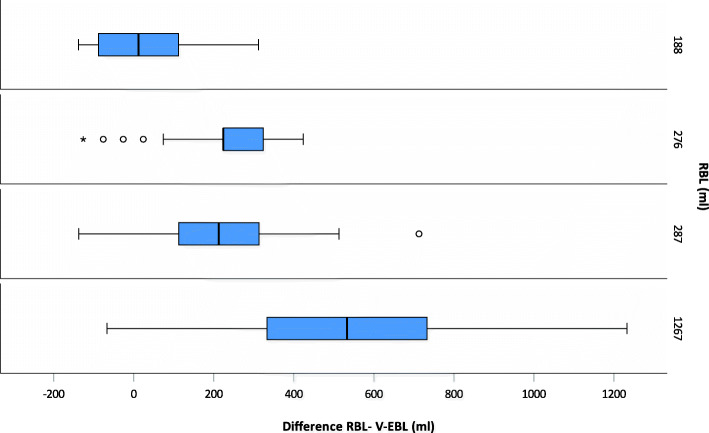
Boxplots for differences between the V-EBL and RBL for canisters Boxplots show differences between the visual blood loss estimate (V-EBL) and the reference blood loss (RBL) for canisters

Figure 5 shows the differences between V-EBL and RBL for canisters per scenario.

**Fig. 6:**
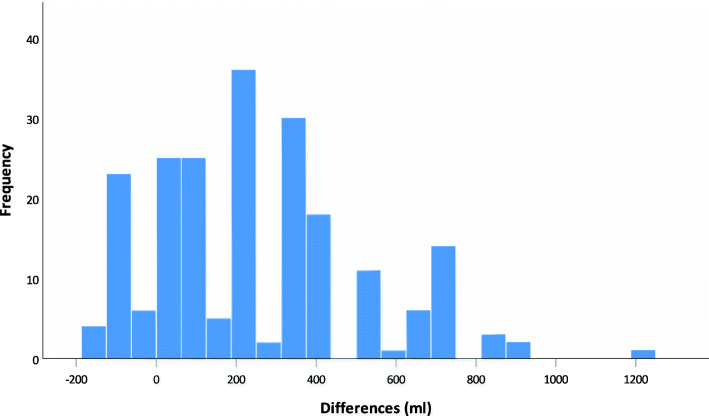
Histogram of the differences The histogram shows frequency of differences between visually estimated (V-EBL) for canister and reference blood loss

The frequency of the respective deviation in visually estimated blood loss from the RBL is shown in the histogram (Fig. 6).

Results from univariate nonparametric confirmation statistics with paired Wilcoxon test regarding visual estimation of canisters show that the deviation of the V-EBL was significant (z = − 10.95, *p* < 0.001, *n* = 220).

**Fig. 7:**
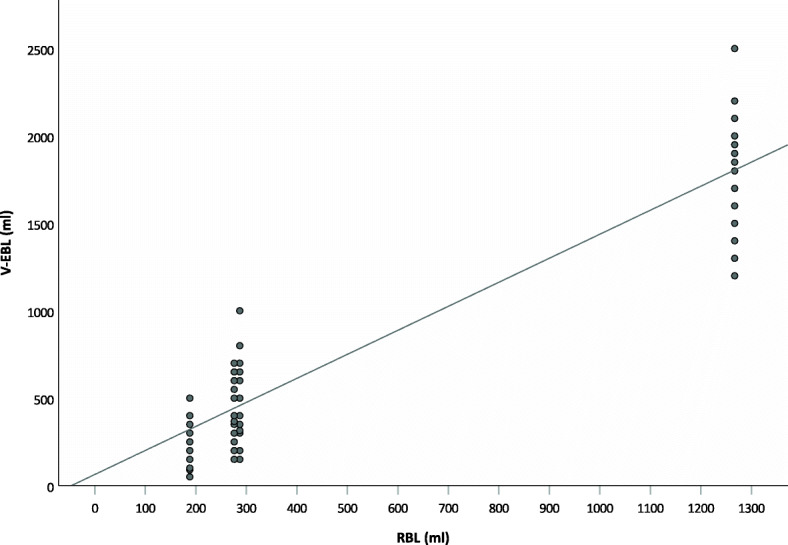
Scatter plots with corresponding regression lines for canisters Scatter plots for visually estimated blood loss (V-EBL) for canisters and corresponding univariate linear regression line as a function of reference blood loss (RBL)

**Fig. 8:**
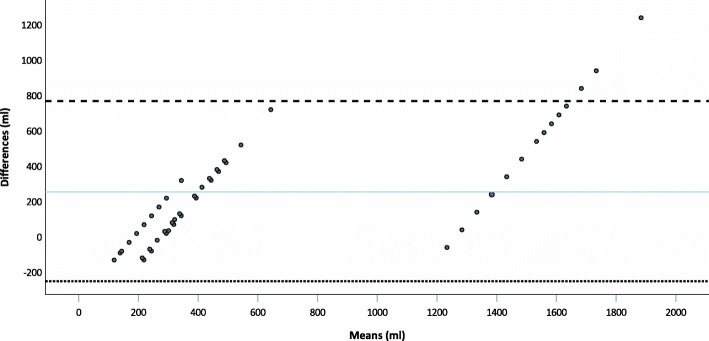
Bland Altman plots for visually estimated blood loss (V-EBL) Bland Altman plots for visually estimated blood loss (V-EBL) for canisters compared to the reference blood loss (RBL) The Bland-Altman plot shows the mean differences (blue line) and the agreement interval within 95% of the differences (bottom: line; top: line)

The visual estimation for canisters correlated moderately with RBL (Spearman’s rho: 0.818; p < 0.001). Scatter plots with corresponding regression lines illustrate the dependence (Fig. 7). Bland-Altman plots of the differences between V-EBL and RBL are shown in Fig. 8.

## Discussion

In this study, we were able to show that the visual measurement of blood loss only moderately correlated with the actual blood loss collected in suction canisters. Both, the univariate analysis and the Bland Altman analysis revealed relevant deviations which are of great clinical relevance.

On the median, the estimated blood loss was greater than the median RBL. Especially for small volumes, the overestimation was higher than for larger volumes. The presented deviations added up to 1233 ml per scenario and were therefore severe and of highest clinical relevance. *Why is knowledge about blood loss relevant?* Intraoperative volume and transfusion management is of great importance for patient safety. Avoiding blood loss and thus preventing transfusions is a core element of Patient Blood Management [7–9]. The indication for a transfusion should be set by individual transfusion triggers. Here, the consideration of blood loss is a crucial factor.

We therefore strongly discourage the use of visual blood loss estimates. Currently, however, V-EBL is the most common method for measuring intraoperative blood loss [1].

The accuracy of measurement using a scaled canister depends on the degree of dilution. In our study we established Hb values between 4.9 and 6.2 g/dl by dilution, comparable to the cases of massive bleeding with little dilution. Internal measurements in the operating room have shown a high variance of Hb values in suction canisters. Under use of high amount of irrigation fluid, e.g. in orthopedics, Hb values as low as 0.5–1.0 g/dl were observed.

*What implications do our results have?* The recording of blood losses is complex. Clinical decisions are rarely made based on only one piece of information. In particular, volume and hemotherapy should be based on multiple factors and primarily on physiologic transfusion triggers. Especially in prolonged operations with continuous blood loss, correct recording of blood loss is an essential component, since Hb, etc., will only change with adequate volume replacement. Losses in canisters, drapes, operating theatre areas and even the floor must be calculated. However, the largest amount of blood usually gets into the suction canister, so that the canisters have a more important role to play. This has not yet been sufficiently taken into account in the research.

*What other alternatives can be used?* A further option is to calculate the blood loss using various formulas based on laboratory parameters such as Hb levels. This can provide an approximation of the bleeding situation [10, 11]. These formulas assume normovolemia. In case of (iatrogenic) dilution results may lead to false results. Furthermore, the volume effects of intraoperative volume therapy, especially with colloidal fluids or plasma, are not considered in these formulas. Therefore, these methods can only serve as a rough approximation in the intraoperative setting. In contrast, the calculation of Hb mass loss is superior to the usual formulas for estimating blood loss, since factors such as dilution have no influence here. It must be considered that such formulas do not allow real-time monitoring of blood loss.

In a meta-analysis [2] on the techniques of intraoperative blood loss recording by our research group, we were able to identify an advantage in measurement of blood volume in sponges by technically supported methods such as colorimetric blood loss estimation. Experimental studies of this system have also been published for canisters [12–18]. With colorimetric blood loss estimation, blood volume in sponges or canisters can be measured in real time. By taking images of sponges or canisters with a smartphone and using colorimetric image correction, the procedure can estimate the blood loss by calculating the loss of Hb mass based on the preoperative Hb value. It also provides real-time information on blood loss and potentially improves the treatment of bleeding patients and targeted hemotherapy.

### Limitations

Based on the fact, that this scenario is a simulation, the usual case details and impressions from the operating theatre are not available for evaluation. There was no interdisciplinary exchange within the surgical team that normally takes place during surgery, e.g. statements from surgeons about acute extreme bleeding or vascular injuries that are included in the anesthetist’s assessment of V-EBL. The participants evaluated a spot check of a normally dynamic bleeding scenario. In a real clinical setting, canisters contain not only diluted blood, but also color- and consistency-changing fluids such as bile, pus, and intestinal contents. CPD-infused blood may have different color and flow characteristics than fresh blood. 360° views of the scenarios were not available, so the participants evaluated the blood loss based on a frontal view of the canisters.

As a simulation is an artificial situation and the Hawthorne effect must be taken into account. It describes that participants as subjects of a study change their behavior. Only a small range of blood volumes and dilutions in canisters was simulated. During the simulation, the canisters were not attached to the suction cup and were therefore not evaluated under vacuum by the participants.

## Conclusion

The discrepancies between the visual estimate of canisters and the actual blood loss are enormous despite the given scales. Therefore, we do not recommend V-EBL in scaled suction canisters. Colorimetric blood loss estimation could be a more accurate option.

## Data Availability

The datasets used or analyzed during the current study are available from the corresponding author on reasonable request.
